# The Effects of Virtual Caregiver Coaching in Antigua & Barbuda on the Implementation of EMT Language Support Strategies in Naturalistic Environments

**DOI:** 10.5195/ijt.2023.6586

**Published:** 2023-12-12

**Authors:** Sensemillah Z. Peters, Klaire Mann Brumbaugh, Amanda Blackwell

**Affiliations:** 1 Speech-Language Pathology Program, Rocky Mountain University of Health Professions, Provo, Utah, USA; 2 Fontbonne University, Clayton, Missouri, USA; 3 Minot State University, Minot, North Dakota, USA

**Keywords:** Caregiver coaching, Communication strategies, Early intervention, Telepractice

## Abstract

This single-case multiple baseline design investigation set out to determine the effectiveness of using a telepractice service delivery model to coach caregivers in Antigua & Barbuda in the use of Enhanced Milieu Teaching (EMT) language support strategies with a child with language impairment. A slightly modified version of the Teach-Model-Coach-Review (TMCR) method was used during virtual instruction to train a caregiver on the language support strategies of environmental arrangement, matched turns, expansions, and time delay with milieu prompting. The caregiver attended sessions three times a week for up to 45 minutes for four weeks. The results of this study indicated a positive relationship between the intervention and caregiver use of strategies. The caregiver demonstrated increased responsiveness to the child's communication attempts and exhibited the use of language support strategies across activities. This study suggests that telepractice can be an effective service delivery model for providing coaching to caregivers.

Situated in the Caribbean is Antigua and Barbuda, twin islands that are highly underserved when it comes to special education and speech-language services (Economic Commission for Latin America and the Caribbean [ECLAC], 2018). The country currently has four special education programs: School for the Deaf, Adele School for children with mild to severe disabilities, Victory Centre of Antigua, and Unit for the Blind and Visually Impaired, which provides support for students in mainstream schools (ECLAC, 2018). There is currently only one speech-language pathology clinic on the island, the Center for the Holistic Advancement of Therapeutic Services (CHATS), established in 2016 and expanded to provide early intervention (EI) services in 2018 (Ambrose, 2020). Approximately three in every 1000 children ages 0–4 years living in Antigua and Barbuda have a speaking disability (ECLAC, 2018), leaving many parents to fend for themselves when their children are identified as late talkers or language impaired (LI). Not only do parents not have enough resources available, but they have not been taught how to provide appropriate language stimulation to their children. Children with LI are at an increased risk for persistent long-term negative outcomes in language skills, literacy development (both reading and writing), social-emotional development and school readiness (Bellom-Harn et al., 2020; DeVeney et al., 2017; Ellis & Thal, 2008; Evans & Brown, 2016; Hatcher, 2018; Lindsay & Strand, 2016). Early intervention (EI) programs connect parents with licensed Speech-Language Pathologists (SLPs) who assess language skills, then collaborate with parents and caregivers to utilize a family centered approach in naturalistic environments, that facilitates language acquisition. In EI, caregivers are the primary interventionists, allowing for more opportunities to practice newly learned skills (Vismara et al., 2012). With advances in technology, caregivers have access to internet and telecommunication technologies, and SLPs can provide services virtually. This is especially important for people who live in underserved areas like Antigua and Barbuda. Parents who cannot get traditional speech therapy services may benefit from remote instruction to better assist with language development.

## Parent-Implemented Interventions

The main goal of parent-implemented interventions is to teach parents how to take the primary interventionists' role, using language support strategies and models to increase responsiveness in naturalistic settings, like routines and child-preferred play-based activities (Akemoğlu et al., 2020). In its infancy, research on parent-implemented intervention was focused on parent responsiveness when facilitating pragmatic language skills, including, but not limited to, joint attention, imitation, and play (Oosterling et al., 2010; Siller et al., 2013). With the successes of social communication, programs shifted to include the study of parent-implemented intervention on parent responsiveness and its effects on language outcomes for children with expressive and receptive language delays (Kaiser & Roberts, 2013; Stoner et al., 2013; Weitzman, 2013). A growing number of research studies show the positive effects of caregiver involvement on achieving parent (language support strategy use) and child (increased language use) outcomes (Hatcher et al., 2019; Roberts & Kaiser, 2011; Roberts et al., 2014).

### Parent-Implemented Enhanced Milieu Teaching

Enhanced Milieu Teaching (EMT) is the most common parent and caregiver-implemented language intervention used in the EI population (Roberts & Kaiser, 2011; Kaiser & Roberts, 2013; Roberts et al., 2014). EMT is defined as a naturalistic language intervention that uses a combination of social and behavioral approaches to early language, using the child's interests and initiations in everyday contexts as opportunities for modeling and prompting language (Roberts et al., 2014; Sheldon & Rush, 2001). EMT consists of four language support strategies (a) matched turns, (b) expansions, (c) time delays, (d) prompting. These strategies are taught in four phases: teaching, modeling, coaching, and reviewing or reflecting (Roberts et al., 2014). These naturalistic intervention strategies support social communication, as the strategies are embedded in a variety of routines throughout the home, school, or community daily, allowing for repeated instruction and increased opportunities for generalization of skills (Kashinath et al., 2006; McDuffie et al., 2013; Meadan et al., 2016; Sheldon & Rush, 2001).

There is a significant amount of research that has been conducted to study the effectiveness of EMT across a wide variety of populations, including but not limited to late takers, children at risk for LIs, children who are deaf and hard of hearing, and children with autism spectrum disorders (ASD; Kaiser & Hancock, 2003; Wright & Kaiser, 2017). The studies demonstrate success in parents learning and implementing naturalistic language interventions, with positive effects on language skills in the child and increased positive effects in parent-child interactions (Romski, 2011). Parent-implemented interventions were also effective in increasing parent knowledge and confidence (Othman & Aziz, 2019; Stredler-Brown, 2017) and parent responsiveness to their children's interactions (Meadan et al., 2016).

Hatcher et al. (2019) conducted a study in Guatemala that looked at caregivers' ability to implement language support strategies for children in an orphanage. There were two caregiver-child dyads for the duration of the study. At baseline, no adult demonstrated the use of targeted language strategies, but immediately following caregiver training and coaching, both adults demonstrated an increase in their use of language support strategies. The adults were then paired with other children, with whom they also demonstrated the use of the trained language support strategies at a level above criterion, thus showing generalization of the learned skills. Although child behaviors were not formally measured during this study, a noticeable improvement in language skills was observed (Hatcher et al., 2019).

Roberts and Kaiser (2011) determined that parent-implemented intervention had significant effects in improving expressive and receptive language skills, evident by increases in vocabulary, grammar, and communicative initiations. Parents who participated in training programs were more likely to increase their use of language support strategies and responsiveness to their child's communication attempts. Not only is this intervention approach effective for late talkers, but it is also effective for children with intellectual disabilities (Roberts & Kaiser, 2011). It has also been shown that active parent participation during parent-implemented therapy sessions correlates with reduced stress levels in parents, which directly improves the quality of parent-child interactions (Guðmundsdóttir et al., 2019).

Parents who have participated in parent-implemented intervention training have shown increased use of environmental arrangements, modeling, expansions, and time delay (Akemoğlu et al., 2020; Guðmundsdóttir et al., 2019; Kaiser & Roberts, 2013; Meadan et al., 2014; Vismara, 2012). Parent-implemented interventions were also effective in increasing parent knowledge and confidence (Othman & Aziz, 2019; Stredler-Brown, 2017) and parent responsiveness to their children's interactions (Meadan et al., 2016).

## Telepractice

With many families living in geographic areas with limited access to EI and allied health services, the use of technology for service delivery can bridge the gap (Meadan et al., 2016). Telepractice requires the use of basic equipment that most families already have in their homes (Snodgrass et al., 2017; Vismara et al., 2012). Families in Antigua and Barbuda (Pan American Health Organization [PAHO], 2012) have access to internet technologies of varying speeds, thus increasing the accessibility of speech therapy services. The COVID-19 pandemic resulted in the immediate need for a change in service delivery models for all health professionals due to lockdowns (Simacek et al., 2020), which ultimately fast-tracked the use of telepractice for speech-language intervention and successfully highlighted the benefits of telepractice (Simacek et al., 2020; Stredler-Brown, 2017). Telepractice allows timely delivery of services, flexibility (time and location), and reduced cancellations (Stredler-Brown, 2017). Studies have shown that parents can receive virtual coaching on language support strategies and implement in-home intervention successfully (Daczewitz et al, 2020; Meadan et al., 2016).

Telepractice provides a switch in the role of the primary interventionist. In this service delivery model, caregivers now directly implement the intervention, as they are trained and coached through strategies by the SLP. The physical absence of the SLP encourages parents to problem solve and apply the strategies taught in previous sessions. Compared to face-to-face services, parents usually take a more passive approach and heavily rely on the SLP to provide direct child intervention (Hamren & Quigley, 2012; Meadan & Daczewitz, 2014). Telepractice thus alleviates many of the challenges (access to available SLPs, scheduling difficulties, travel times, and associated costs) faced by both parents and SLPs for home-based EI services. Parents who have used teletherapy services state that it is comparable to traditional in-person services (Vismara et al., 2012). It is an acceptable, feasible, and effective way to access speech-language intervention (Akemoğlu et al., 2020).

Meadan et al. (2016) studied the effectiveness of coaching on parent use of language strategies and child communication via telepractice, by modifying the Parent-Implemented Communication Strategies Program (PiCS). Three parent-child dyads were trained and observed using a multiple-baseline design examining three conditions, (a) environmental arrangement and modeling, (b) environmental arrangement and mand-model, and (c) environmental arrangement and time delay. The results varied, but there were increases in the rate and quality of strategy use during the coaching phase. The use of strategies across all participants diminished from coaching to maintenance; however, the use of strategies was better when compared to baseline use. The children in the study were more responsive during the coaching phase, noted by the increase in initiations (Meadan et al., 2016). Ultimately, the results indicated that teletherapy as a service delivery model for EI services was effective.

Daczewitz et al. (2020) employed the use of PiCS to study the effects the program had on a parent of a child who was deaf and hard-of-hearing (DHH) when the instruction was presented virtually through a computer and smartphone. Results from the study showed that the parent increased the use of language support strategies (environmental arrangement, modeling, mand model and time delay). The parent also used simpler language, which positively affected the child's communication attempts as the child initiated and responded more (Daczewitz et al., 2020).

## Purpose of the Study

The purpose of this study was to investigate the effects of virtual instruction on caregiver's use of EMT strategies with children 24–36 months with a language impairment. Many of the studies that have been conducted on caregiver-implemented interventions have been completed in the United States (Brown, 2015; Daczewitz et al., 2020; Fairweather et al., 2016; Kaiser & Roberts, 2013; Meadan et al., 2014; Meadan & Daczewitz, 2014; Meadan et al., 2013; Meadan et al., 2016; Roberts et al., 2014; Roberts & Kaiser, 2015; Stoner et al., 2013; Vismara et al., 2012; Weitzman, 2013; Wright & Kaiser, 2017). CHATS is the only speech pathology clinic in Antigua and Barbuda, highlighting the lack of speech-language resources. Telepractice provides a means for families to have access to the services that they need. Therefore, there is a need for the same type of research in other countries in the Caribbean with culturally diverse populations. There are currently no other studies on caregiver-implemented interventions in Antigua and Barbuda or other English-speaking Caribbean countries. This study replicates the coaching strategies cited in Hatcher et al. (2019) used to train caregivers in Guatemala. Other aspects of the investigation were modified including treatment modality, inclusion criteria, and the follow-up phase.

## Method

### Participants

#### Recruitment

A recruitment flier approved by Rocky Mountain University of Health Professional's Institutional Review Board was posted on SpeakEZ Theracare's Facebook page (SpeakEZ Theracare, n.d.). SpeakEZ Theracare is the first author's private practice where services are provided via telepractice to pediatric clients and their families. Recruitment was done through self-selection. Three caregiver-child dyads met the preliminary inclusion criteria. Each caregiver met with the first author via Zoom to discuss and review informed consents for all participants (both caregiver and child), including consent to participate in the study, consent to record sessions and collect data. To assess for the understanding of the informed consent, the caregivers were asked to retell the procedures to the examiner. Each caregiver received a copy of informed consent via email and given 24 hours to consider participation in the study. After signed informed consent was received, the *Receptive Expressive Emergent Language Test - 4th Edition* (REEL-4; Brown et al., 2020) was administered to determine the severity of the child's language impairment. The first author, an SLP and clinical doctoral student, was the sole interventionist for this study. A second SLP was used to gather fidelity and interrater reliability measures.

#### Inclusion Criteria/Exclusion Criteria

Initially, three caregiver-child dyads expressed interest in the study. One child was excluded due to the assessment scores falling outside of the inclusion criteria. The other potential participant was excluded from the study due to illness and the inability to begin the intervention within the specified timeframe.

One caregiver-child dyad participated in this study through self-identification, meeting all of the inclusion criteria. The caregiver, Sarah, was a 55-year-old Afro-Caribbean female with diploma level education. The child, Max, was a 33-month-old Afro-Caribbean boy from a triplet set. The child was born via C-section at 27 weeks gestation and spent 6 weeks in the NICU before being released. Sarah was Max's biological grandmother. Max was considered developmentally healthy except for language development when compared to his siblings, per caregiver report, and peers via standardized assessment. Max does not attend daycare/preschool and is cared for by the primary caregiver in this study. The biological mother, a 37-year-old Afro-Caribbean female, with master's level education, attended all virtual sessions as an observer and completed all study paperwork including consents and follow-up survey.

Max, was included because he (a) was between the ages of 24 months and 36 months; (b) had normal hearing as reported by the parent; (c) had no other medical diagnoses as reported by the parent; (d) used English as his primary language; (e) had an expressive vocabulary of at least five words; (f) had a suspected language delay; (g) not currently enrolled in or receiving speech-language services; and (h) caregivers exhibited interest and willingness to implement strategies. For the purposes of this research, a language delay was classified as scores within 1–1.5 SD below the mean on the REEL-4 assessment. Max had an expressive language score of 80 (below average), a receptive language score of 100 (average), and a total ability score of 87 (below average).

### Research Design

A single-case multiple-baseline across behaviors was used to evaluate the effectiveness of parent-implemented EMT strategies on caregiver's use of language support strategies in a 33-month-old child with a language delay. The measured behaviors were environmental arrangement, matched turns, expansions, and time delays with milieu prompting. EMT strategies were taught through telepractice using the slightly modified version of the teach-model-coach-review (TMCR) model used in earlier research (Roberts et al., 2014). The *teach* component introduced the language strategies. The *model* component in this study consisted of illustrations with video examples of the language strategies. In the original approach, the SLP interacts directly with the child modeling the strategy. *Coaching* allowed the caregiver to apply the knowledge gained with real-time feedback. The *review* component facilitated reflection and evaluation of strategies used and review of recorded sessions. Data collection sheets and fidelity checklists used throughout the study were adapted from previous work in this area (Roberts et al., 2014 & Hatcher, 2019).

### Setting and Materials

The first author used a MacBook computer and the Health Insurance Portability and Accountability Act (HIPAA; American Speech-Language-Hearing Association, n.d.) compliant version of ZOOM video conferencing software for baseline, intervention, and follow-up sessions. Sessions were conducted from the investigator's home office. Sarah, used a laptop for all baseline, training, and coaching sessions, except one where she used a phone. Sarah utilized a variety of child-preferred, age-appropriate toys, including cars, trucks, blocks, puzzles, books, and a dressing and snack routine to practice the skills. Each session took place in a bedroom within the home, with the door closed to minimize distractions, with the computer/phone positioned either on the bed or the dresser.

### Procedures of the Investigation

#### Baseline

Baseline measurements of the use of environmental arrangement, matched turns, expansions, and time delays with milieu prompting were obtained through three 10-minute samples of caregiver-child interaction, using three different toys (flashcards, puzzles, and blocks) of choice during a single session using direct observation. Baseline stability was obtained during one session with three observation periods. Stability was considered met when 50% of the data points fell within 50% of the mean (Alberto & Troutman, 1982). During baseline sessions, the frequency and type of language support strategy were recorded in their natural environment without teaching or coaching.

#### Intervention

The intervention phase lasted four weeks for a total of 12 sessions. Each week one new EMT language support strategy was introduced. Session one was the teach/introductory session, where Sarah was taught the definition, rationale, and implementation procedures of that week's strategy using PowerPoint slides and video examples. Teaching sessions began with three minutes of free play recorded to collect data on the use of strategies taught and strategies not yet introduced.

Following the teaching of a new strategy, Max engaged in a 10-minute practice session where Sarah was coached to implement the use of the new strategy. The investigator provided feedback throughout this interaction, and Sarah was encouraged to ask questions and review the information provided. The teaching session was followed by two 30-minute coaching sessions later that week. Sarah was not provided with handouts as a point of reference during the study, thus relying only on the information presented during teaching and coaching sessions. Additionally, there was no criterion requirement prior to the introduction of a new strategy. The lengths and structures of both training and coaching sessions are detailed in Table 1.

**Table 1.** Description of Length and Structure of Intervention Sessions

Training Session (1^st^ session each wk.)	Coaching Session (2 subsequent sessions each wk.)
3 min data collection during “free play” (child needed)	3 min data collection during “free play” (child needed)
15 minutes of education using videos, PowerPoint presentations	5–7 minutes to converse about anything that may have transpired since the last session, answer any questions.
10 minutes for practice and feedback (child participation needed)	15 minutes for practicing language strategies across selected events/routines, with specific reinforcement and feedback provided throughout the session. (child participation needed)
5–7 minutes for reflection and carryover	10–15 minutes for reflection and carryover

Following each session, Sarah was instructed to use the strategies across various routines and activities in the natural learning environment until the next scheduled session. Generalization of previously learned skills was assessed during subsequent sessions. At the end of the study, Sarah was provided with handouts in the form of PowerPoint slides with information about the language support strategies used in the investigation for future reference.

### Variables

The independent variable for this investigation was coaching provided to the caregiver for the four EMT language support strategies. Coaching involved all the components of the TMCR approach. The dependent variable in this investigation was the frequency of caregiver's implementation of these strategies.

### EMT Strategies

#### Environmental Arrangement

Environmental arrangement is the physical manipulation of the immediate environment by the caregiver to increase opportunities to elicit child communication (Roberts et al., 2014), using the procedures of *in view but out of reach, needing assistance*, and *being silly* (Hatcher, 2019)*.* For the *in view but out of reach* arrangement, Sarah was encouraged to place desired items where the child can see them but not reach them. In the *needing assistance* arrangement, Sarah was taught to use toys or materials that the child cannot use on their own (e.g., wind-up toys). For the *being silly* arrangement, Sarah was taught to use toys or materials in a manner that is different from the typical use. Sarah was taught that the rationale for arranging the environment is to manage distractions and challenging behaviors, increase and improve the quality of play interactions with the child, and increase opportunities for language learning and language use (Hatcher, 2019).

#### Matched Turns

A matched turn is the caregiver's verbal or nonverbal conversational turn, immediately following the child's communicative attempt (Roberts et al., 2014). Sarah was taught to copy Max's actions, sounds, and words. Turns were matched by showing Max a different way to play with the toy or introducing a new sound/word associated with the context. The turns between Max and Sarah did not need to be identical, but they must be equal in number.

#### Expansions

Expansions are language support strategies that require the caregiver to add a word or two to the child's original utterance or change a word to make it grammatically correct (Roberts et al., 2014). Sarah was taught to expand by imitating the gesture, vocalization, or utterance by adding a word or two, not exceeding Max's level. For example, if Max produced a three-word utterance, the expansion utterance should not exceed five words.

#### Time Delays with Milieu Prompting

A time delay is a nonverbal attempt by the caregiver to elicit a verbal response or a more complex response from the child (Roberts et al., 2014). Sarah was instructed to wait up to five seconds and withhold the item of interest before proving a verbal prompt. If there is no response, present Max with choices (e.g., “ball” or “blocks”) and wait for him to respond. If Max uses a gesture (pointing or eye gaze), Sarah should provide the verbal label and give the item to Max. Milieu prompting is a verbal language support strategy where the caregiver uses a sequence of prompts in response to the child's verbal or nonverbal request (Roberts et al., 2014).

### Follow-up Session

One-week post-intervention, the caregiver-child dyad attended a follow-up session to assess for generalization and maintenance of language support strategies. The session mirrored the protocol from baseline and lasted 10 minutes, with no coaching or feedback provided.

### Interrater Reliability

A licensed speech-language pathologist completed the fidelity checklists and the data collection forms at the end of the investigation. Prior to intervention the SLP (second-rater) was trained using practice videos to identify the use of language strategies between caregiver-child interactions, and coding to complete data collection sheets. Sessions were randomly selected using Research Randomizer and sent to the second-rater via encrypted email. Interrater reliability was calculated by dividing the number of agreements and the total number of ratings. The second rater viewed 5/12 (42%) intervention sessions, baseline, and follow-up sessions, obtaining an interrater reliability score of 85.37%, surpassing the recommended 80% (Ledford & Gast, 2018).

### Procedural Fidelity

The second rater used checklists to evaluate the fidelity of implementing all components of the TMCR model during coaching and feedback sessions after viewing 42% of recorded sessions (Ledford & Gast, 2018). Procedural fidelity was calculated at 100% for teaching sessions and 90% for coaching and feedback sessions.

### Social Validity

After the study, the parent was sent a link to complete an eight-item questionnaire using a combination of a 5-point Likert Scale and open-ended questions about (a) their perceptions of the program; (b) their understanding of language support strategies; (c) feasibility of the program; (d) ease of access; (e) barriers with technology; (f) likelihood of using the language support strategies; (f) changes they would make to the program; (g) likelihood of recommending the program to others.

### Data Analysis

Data analysis was completed using a combination of descriptive statistics and visual analysis. Caregiver behaviors were measured using event recording of the occurrence of all target behaviors. Three-minute data collection periods were recorded and analyzed by the first author and second-rater. Data were plotted on a graph and analyzed for trends and levels of change. The first author also utilized an investigator's journal to document confounding factors that may have contributed to a downward trend. Treatment is considered effective if there is a 20% increase from baseline at the end of the intervention phase (Ledford & Gast, 2018).

## Results

### Caregiver Behaviors

**Figure 1.**
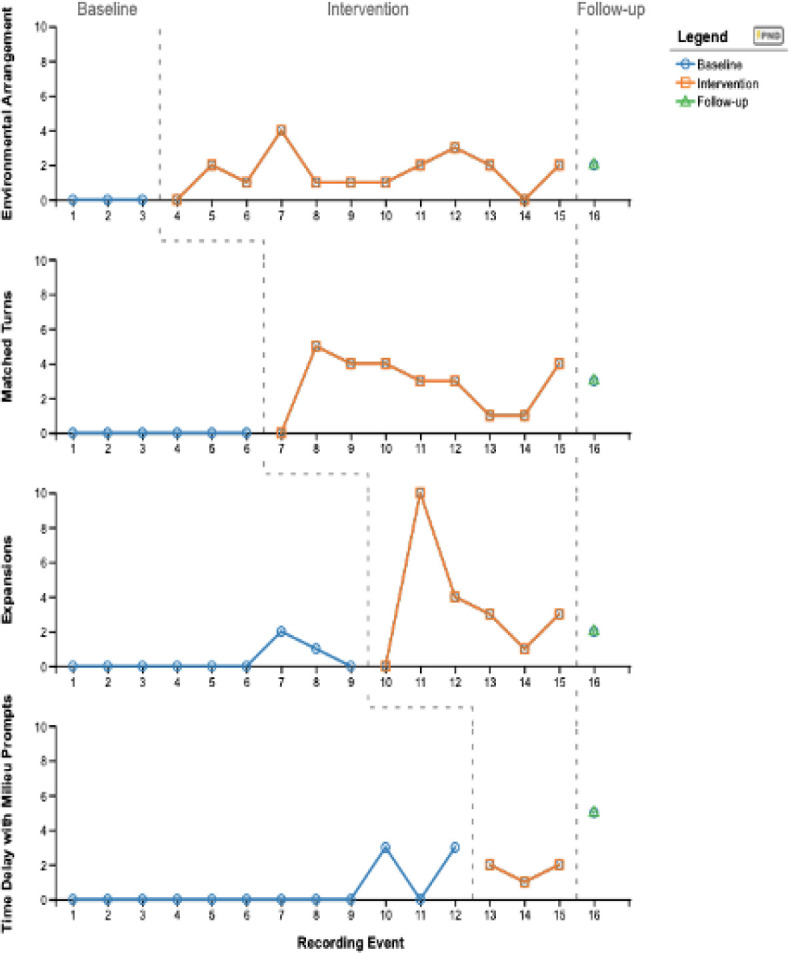
Number of Times Caregiver Correctly Used Language Support Strategies

Results are illustrated in Figure 1 and represent the frequency of caregiver use of language support strategies during baseline, intervention, and follow-up sessions. Sarah participated in 14 sessions (one baseline session with three recording events, four training sessions, eight coaching sessions, one follow-up session). During the baseline session, Sarah did not demonstrate the use of environmental arrangement or matched turns; but expansion and time delay with milieu prompting were observed. Sarah used puzzles, flashcards, and blocks for the interactions and demonstrated frequent use of questions - “What is it?” “What color is it?” “What shape is it?” - only allowing one-two seconds before repeating the question. Interactions were structured and pre-academic in nature.

#### Environmental Arrangement

The baseline phase consisted of a single session with three data recording events, followed by three intervention sessions, then maintenance. The baseline was 100% stable. The percentage of non-overlapping data (PND) between baseline and intervention was 83% (10/12 data points), indicating a noticeable positive effect of the intervention on caregiver behavior. Eleven data points fell above the two standard deviation (SD) band, also indicating a significant change from baseline to intervention. Sarah demonstrated the use of environmental arrangement across intervention phases, on average 1.62 times (range zero to four times) after learning this language support strategy. During the follow-up session, Sarah used environmental arrangement two times indicating maintenance of strategy and a clinically significant change.

#### Matched Turns

The baseline phase for this language support strategy extended the previous condition by three additional sessions. This baseline was also 100% stable. The PND between baseline and intervention was 89% (8/9 data points), indicating a noticeable positive effect of the intervention on caregiver behavior. Nine data points fell above the two SD band, also indicating a clinically significant change from baseline to intervention. Sarah demonstrated the use of matched turns across intervention phases, on average 2.8 times (range zero to five times). A single follow-up data point of three was observed, indicating a decrease in the use of strategy after the completion of the study.

#### Expansions

The baseline phase for expansion consisted of nine recording events. The baseline for this intervention phase was unstable because more than 50% of the data points fell below the mean. Although the baseline was unstable, because the caregiver demonstrated use of this strategy twice, prior to it being taught, the intervention proceeded. The PND between baseline and intervention was 67% (4/6 data points), making it difficult to state whether the change in behavior was a direct result of the intervention. Sarah demonstrated use of this strategy four times more than during baseline, with a range of zero to ten. A single follow-up data point of two was observed, indicating a decrease of one in caregiver behaviors.

#### Time Delay with Milieu Prompting

There were 12 recording events in the baseline phase for this strategy. Baseline stability was not achieved because 50% of the data points fell below the mean, but the intervention continued. The PND between baseline and intervention was 0% (0/3 data points), indicating no difference in the effectiveness of the intervention on caregiver behavior. Although data shows no difference in the intervention phase, a single follow-up data point of five was observed, indicating an increase in caregiver behaviors.

### Social Validity

During the *review* component of TMCR, Sarah expressed that implementing three to five second wait time was a personal struggle that she had not realized until participating in this study. As the intervention progressed Sarah learned to use fill-in-the-blank vs. open question prompts, which was more effective in allowing the appropriate wait time. Sarah also reported that matched turns was a difficult strategy to implement during play, evident by missed opportunities to follow Max's lead, often requiring reminders during the coaching sessions. Expansions and environmental arrangements were reported to be the easiest strategies to implement and generalize. Sarah's responsiveness to Max's communication attempts increased, improving reciprocal verbal communication. Sarah also increased her communication with Max as they engaged in play, by describing what she was doing and what Max was doing, which provided exposure to additional language.

The parent answered positively to all questions on the post-intervention survey. Table 2 contains the questions and responses. Sarah communicated that participation in this study had changed the language used throughout the home environment, not only with Max but also with his sisters. The flexibility of telepractice allowed the child's mother to attend sessions and learn strategies, even though Sarah was the primary caregiver.

**Table 2.** Social Validity Questionnaire Results

Parent Satisfaction Survey	
I understood the information presented in this study regarding the language support strategies.	Strongly agree
The length of the sessions was enough to learn and practice the strategies.	Strongly agree
I had no difficulty accessing the online platform for the intervention.	Strongly agree
I will continue to use the language support strategies taught during this study with my child.	Strongly agree
The strategies I learned in this study has improved my interaction with my child.	Strongly agree
What change(s), if any, would you make to the study?	*Nothing I can pinpoint at this moment*
What is the likelihood of you recommending this study to another parent?	Very likely
What are your overall thoughts about the study?	*I think this study was an awesome idea, especially in a pandemic era in which interaction is limited. It is great, involving both a parent and child. This study had significant involvement of parents, ensuring they understood, it takes more than a session for improvement to be seen. It has to involve daily interaction and a conscious effort on our part.*

## Discussion

The purpose of this study was to answer the research question “Can caregivers of children with LI between the ages of 2–3 years residing in Antigua and Barbuda be trained to implement EMT strategies via telepractice?” The results of this study demonstrated that Sarah could learn and use language support strategies with a child with LI, after completing a short-term investigation. This study used a the TMCR method (Roberts et al., 2014) similar to that used by Hatcher (2019) with caregivers in Guatemala. The strategies chosen for this investigation were proven to be the most common for facilitating language development in children with LI (Hatcher, 2019). Results from the current study show that Sarah demonstrated the use of each language support strategy from baseline to intervention, with varying frequencies like the caregiver from the Daczewitz et al. (2020) study. This investigation's findings are consistent with that of previous research that supports caregiver coaching as an effective intervention (Daczewitz, 2020; Meadan et al., 2016; Roberts & Kaiser Kaiser, 2011, Roberts et al., 2014; Stredler-Brown, 2017, Vismara et al., 2012). Furthermore, it supports the use of caregiver coaching through telepractice, as this service delivery model requires more observation and guidance from providers, and more interaction and involvement from caregivers (Stredler-Brown, 2017). Stredler-Brown (2017) compared in-person therapy with telepractice service delivery for infants and toddlers who were deaf or hard of hearing and concluded that telepractice allowed providers to observe more of the caregiver-child interactions and provided more parent practice with feedback to improve the interactions and support language learning.

The data represented in the graphs were collected during the first three minutes of each session and did not reflect Sarah's performance during the coaching sessions. Throughout the intervention Sarah demonstrated the use of language strategies more frequently with feedback than she did without feedback. Feedback was provided during coaching sessions when Sarah missed opportunities to demonstrate the use of target behaviors. This highlights a positive correlation between the use of video training and coaching on caregiver use of strategies. The coaching sessions allowed time for self-reflection and feedback, and Sarah demonstrated increased understanding of the strategies used. When an activity did not go as planned, Sarah was able to walk through the steps and explain the expected behavior. As the intervention progressed, Sarah was also able to identify moments where she missed an opportunity to use one of the learned strategies while watching recorded sessions.

During baseline and the beginning weeks of the intervention, Sarah exhibited the overuse of consecutive questioning without wait time (e.g., *What color is it? What shape is it? Where does the pants go?*), but with coaching this behavior was reduced as she began to implement the use of time-delay and milieu-prompting (e.g., *pants go on my….” “The heart is …” “is it red or blue”*). The use of different prompts provided better opportunities for Max to use words to communicate. The results of this study is like that of Meadan et al. (2016), where the caregivers were taught and coached through telepractice, showing a reduction in the use of yes/no questioning to more open-ended question prompts to increase child communication.

It was noted that Sarah struggled to match turns with Max which was evident in the range of frequencies (zero to five times) graphed. Sarah was adamant about completing all planned activities, sometimes prematurely switching the activity when Max was still interested in the previous activity. This then resulted in a power struggle and reduced communication and engagement from Max. There was no formal teaching of pre-verbal skills such as joint attention, knowing how and when to join in play, and picking appropriate toys prior to the start of intervention. These are important components that teach caregivers how to connect and engage with the child prior to teaching language (Hatcher et al, 2019; Roberts & Kaiser, 2011). Sarah could have benefited from this explicit training. Another issue observed was the presentation of too many options (coloring book, crayons in closed container, blocks, and puzzle with pieces in a Ziploc bag). Max had a difficult time focusing on one task, often shifting attention to another item, resulting in Sarah trying to reengage him without independently taking advantage of the opportunities to use any of the language strategies. This is reflected on data point 14 with a decrease in the use of all language support strategies.

Max exhibited increased engagement when using puzzles, dinosaurs, and book reading. Sarah demonstrated an increase in language by using various word types (nouns, adjectives, and verbs) to talk about what she was doing, and what Max was doing. Sarah used simple utterances exhibiting the expansion strategy. For example, Max said, “*dinosaur jump,”* and Sarah responded, “*blue dinosaur is jumping.”* Through self-reflection Sarah revealed that expansion strategy was one of the easier strategies to implement and was seen more outside of the data collection period. Similar comments were made by the participants in the Hatcher et al., (2019) study, where they also found expansion to be an easier strategy to implement. The shared book reading activity provided increased attention and engagement from Max. Sarah exhibited increased use of expansion, open prompts, wait time and responsiveness to Max's communication attempts. Sarah's increases in communication provided a verbal model for Max.

This study extends previous research on caregiver-implemented language interventions in culturally and linguistically diverse countries (Hatcher, 2019) using internet technologies in their homes (Daczewitz, 2020; Meadan et al., 2016; Roberts et al., 2014; Stredler-Brown, 2017, Vismara et al., 2012), by being the first study to use EMT strategies in an English-speaking Caribbean Island. The use of internet technology shows that a service not readily available in Antigua and Barbuda can be provided to families in need, as caregivers are able to learn strategies.

### Strengths of the Investigation

The results from the current study indicate that a virtual caregiver training program using EMT language support strategies may be effective in increasing caregiver use of strategies during semi-structured play activities. The service delivery model was one of convenience for both the caregiver and service provider, allowing for the caregiver to attend all three sessions weekly even when rescheduling was needed. The use of videos embedded in training and coaching sessions were deemed helpful by the caregiver, as it “tied” in the information that was presented verbally.

### Limitations of the Investigation

Although the results were favorable and add to the body of research on caregiver-implemented intervention with families in culturally diverse countries, there are a few limitations that should be considered. One limitation was the number of participants. Since there was only one participant, it cannot be determined that other caregivers would have responded the same way. This eliminated the opportunity for comparison and analysis to determine the true effects of the study. A second limitation was the length of the study. The caregiver could have benefited from longer intervention with more coaching, as Sarah demonstrated increased use of strategies with feedback. More time may be needed to increase independent use of these strategies. A third limitation was no explicit teaching in pre-verbal skills such as joint attention, child-directed learning, toy selection, and play skills. A fourth limitation noted was connectivity and positioning issues during some sessions, making it difficult to view the entire interaction and provide coaching. Using multiple devices or a single device that captures more space would be beneficial in future studies. A fifth notable limitation was the overlapping of strategies. While teaching both matched turns and expansions, time delay was prematurely introduced to give the child time to process and respond to adult communication.

### Implications for Future Research

The aforementioned limitations suggest modifications for new investigations. Future research should consider the use of headphones for the caregiver to reduce distraction during the session and encourage a more natural interaction. This removes the child's ability to hear and see the speech-language pathologist, thus enhancing the coaching experience from their caregiver. Additional research is needed to assess maintenance and generalization of skills across various activities of daily living that are both structured and unstructured, with more participants and longer intervention. An increase in the number of dyads would allow for the effect of the intervention to be replicated and increase the external validity of the results. Finally, this investigation assessed the changes in caregiver behavior and did not evaluate the change in clinical presentation of the child. Future studies should evaluate if these strategies yielded gains in the child's language skills.

## Conclusions

The results indicate that a virtual caregiver-implemented language intervention program may be effective in teaching caregivers of young children with language impairment during semi-structured activities in the home environment. Even though there were several limitations to the study, the claim was proven that caregivers could learn and implement the use of language support strategies throughout activities of daily living. Given modifications for future research, more information can be obtained. This study adds to the existing literature supporting caregiver-implemented intervention via virtual instruction in culturally and linguistically diverse countries.

## References

[B1] Akemoğlu, Y., Muharib, R., & Meadan, H. (2020). A systematic and quality review of parent-implemented language and communication interventions conducted via telepractice. Journal of Behavioral Education, 29(2), 282–316. 10.1007/s10864-019-09356-3

[B2] Alberto, P. A., & Troutman, A. C. (1982) Applied behavior analysis for teachers. Merrill.

[B3] Ambrose, T. (2020, August 11). Public health spotlight with Mona Gardner. All about the scrublife. https://allaboutthescrublife.com/2020/08/11/public-health-spotlight-with-mona-gardner/

[B4] American Speech-Language-Hearing Association. (n.d.-b). Telepractice. (Practice Portal). www.asha.org/Practice-Portal/Professional-Issues/Telepractice/

[B5] Bellom-Harn, M. L., Morris, L. R., Manchaiah, V., & Harn, W. E. (2020). Use of videos and digital media in parent-implemented interventions for parents of children with primary speech sound and/or language disorders: A scoping review. Journal of Child and Family Studies, 1–13. Advance online publication. 10.1007/s10826-020-01842-x PMC7529088 33024404

[B6] Brown, V. L., Bzoch, K. R., & League, R. (2020). Receptive-Expressive Emergent Language Test – 4th Edition. PRO-ED Inc.

[B7] Daczewitz, M., Meadan-Kaplansky, H., & Borders, C. (2020). PiCs: Telepractice coaching for a parent of a child who is hard-of-hearing. Deafness & Education International, 22(2), 113–138. 10.1080/14643154.2019.1587235

[B8] DeVeney, S. L., Hagaman, J. L., & Bjornsen, A. L. (2017). Parent-implemented versus clinician-directed interventions for late-talking toddlers: A systematic review of the literature. Communication Disorders Quarterly, 39(1), 293–302. 10.1177/1525740117705116.

[B9] Economic Commission for Latin America and the Caribbean [ECLAC]. Disability, human rights and public policy in the Caribbean. LC/TS.2017/151, LC/CAR/TS.2017/12, 2018. https://www.cepal.org/en/publications/43306-disability-human-rights-and-public-policy-caribbean-situation-analysis

[B10] Ellis, E. M., & Thal, D. J. (2008). Early language delay and risk for language impairment. Perspective on Language Learning and Education, 15(3), 93–100. 10.1044/lle15.3.93

[B11] Evans, J., & Brown, T. (2016). Specific language impairment. In G. Hickok & S. L. Small, Neurobiology of Language (1st ed., pp. 899–912). 10.1016/B978-0-12-407794-2.00072-9.

[B12] Fairweather, G. C., Lincoln, M., & Ramsden, R. (2016). Speech-language pathology teletherapy in rural and remote educational settings: Decreasing service inequities. International Journal of Speech-Language Pathology, 18(6), 592–602. 10.3109/17549507.2016.1143973 27063692

[B13] Guðmundsdóttir, K., Sigurðardóttir, Z., & Ala'i-Rosales, S. (2019). Extending caregiver training via telecommunication for rural Icelandic children with autism. Rural Special Education Quarterly, 38(1), 26–42. 10.1177/87568705187835

[B14] Hamren, K., & Quigley, S. (2012). Implementing coaching in a natural environment through distance technologies. The Volta Review, 112(3), 403–407.

[B15] Hatcher, C. A. (2018). Parent-implemented language intervention with young children from low-SES environments who have language impairment. Theses and Dissertations–Rehabilitation Sciences, 45. 10.13023/ETD.2018.006

[B16] Hatcher, C. A., Grisham-Brown, J., & Sese, K. (2019). Teaching and coaching caregivers in Guatemala to promote language in young children. Journal of International Special Needs Education, 22(1), 1–13. 10.9782/17-00021

[B17] Kaiser, A.P., & Hancock, T.B. (2003). Teaching parents new skills to support their young children's development. Infants & Young Children, 16(1), 9–21. 10.1097/00001163-200301000-00003

[B18] Kaiser, A. P., & Roberts, M. Y. (2013). Parents as communication partners: an evidence-based strategy for improving parent support for language and communication in everyday settings. Perspectives on Language Learning and Education, 20(3), 96–111. 10.1044/lle20.3.96

[B19] Kashinath, S., Woods, J., & Goldstein, H. (2006). Enhancing generalized teaching strategy use in daily routines by parents of children with autism. Journal of Speech, Language, and Hearing Research, 49(3), 466–485. 10.1044/1092-4388(2006/036) 16787891

[B20] Ledford, J. R., & Gast, D. L. (2018). Single case research methodology: Applications in special education and behavioral sciences (3rd ed.). Routledge.

[B21] Lindsay G., & Strand S. (2016). Children with language impairment: Prevalence, associated difficulties, and ethnic disproportionality in an English population. Frontiers in Education, 1(2). 10.3389/feduc.2016.00002

[B22] McDuffie, A., Machalicek, W., Oakes, A., Haebig, E., Weismer, S.E., & Abbeduto, L. (2013). Distance video-teleconferencing in early intervention: Pilot study of a naturalistic parent-implemented language intervention. Topics in Early Childhood Special Education, 33(3), 172–185. 10.1177/0271121413476348

[B23] Meadan, H., Angell, M. E., Stoner, J. B., & Daczewitz, M. E. (2014). Parent-implemented social-pragmatic communication intervention: A pilot study. Focus on Autism and Other Developmental Disabilities, 29(2), 95–110. 10.1177/1088357613517504

[B24] Meadan, H., & Daczewitz, M. E. (2014). Internet-based intervention training for parents of young children with disabilities: A promising service-delivery model. Early Child Development and Care 185(1), 155–169. 10.1080/03004430.2014.908866

[B25] Meadan, H., Meyer, L. E., Snodgrass, M. R., & Halle, J. W. (2013). Coaching parents of young children with autism in rural areas using internet-based technologies: A pilot program. Rural Special Education Quarterly, 32(3), 3–10. 10.1177/875687051303200302

[B26] Meadan, H., Snodgrass, M., Meyer, L. E., Fisher, K. W., Chung, M. Y., & Halle, J. W. (2016). Internet-based parent-implemented intervention for young children with autism: A pilot study. Journal of Early Intervention, 38(1), 3–23. 10.1177/1053815116630327

[B27] Oosterling, I., Visser, J., Sophie, S., Rommelse, N., Donders, R., Woudenberg, T., Roos, S., Jan van der Gaag, R., Buitelaar, J. (2010). Randomized controlled trial of the focus parent training for toddlers with autism: 1-year outcome. Journal of Autism and Developmental Disorders 40, 1447–1458. 10.1007/s10803-010-1004-0 20440639 PMC2980624

[B28] Othman, B., & Aziz, N. (2019). Piloting teletherapy for children with hearing loss in Malaysia. Jurnal Pendidkan Bitara UPSI 12, 35–46

[B29] Pan American Health Organization. (2012). Antigua & Barbuda. Health in the Americas: Country Volume. https://www.paho.org/salud-en-las-americas-2012/dmdocuments/hia-2012-ant-barbuda.pdf?ua=1

[B30] Roberts, M. Y., & Kaiser, A. P. (2011). The effectiveness of parent-implemented language interventions: A meta-analysis. American Journal of Speech-Language Pathology, 20(3), 180–199. 10.1044/1058-0360(2011/10-0055) 21478280

[B31] Roberts, M. Y., Kaiser, A. P., Wolfe, C. E., Bryant, J. D., & Spidalieri, A. M. (2014). Effects of the Teach-Model-Coach-Review instructional approach on caregiver use of language support strategies and children's expressive language skills. Journal of Speech, Language, and Hearing Research, 57(5), 1851–1869. 10.1044/2014_JSLHR-L-13-0113 24950492

[B32] Roberts, M. Y., & Kaiser, A. P. (2015). Early intervention for toddlers with language delays: A randomized controlled trial. Pediatrics, 135(4), 686–693. 10.1542/peds.2014-2134 25733749 PMC4379460

[B33] Rogers, S. J., & Dawson, G. (2010). Early Start Denver Model curriculum checklist for young children with Autism. Guilford.

[B34] Romski, M., Sevcik, R. A., Adamson, L. B., Smith, A., Cheslock, M., & Bakeman, R. (2011). Parent perceptions of the language development of toddlers with developmental delays before and after participation in parent-coached language interventions. American Journal of Speech-Language Pathology, 20(2), 111–118. 10.1044/1058-0360(2011/09-0087) 21330651

[B35] Sheldon, M., & Rush, D., (2001). The ten myths about providing early intervention services in natural environments. Infants and Young Children, 14(1), 1–13.

[B36] Siller, M., Hutman, T., and Sigman, M. (2013). A parent-mediated intervention to increase responsive parental behaviors and child communication in children with ASD: A randomized clinical trial. Journal of Autism and Developmental Disorders 43(3), 540–555. 10.1007/s10803-012-1584-y 22825926 PMC3511916

[B37] Simacek, J., Elmquist, M., Dimian, A., & Reichle, J. (2020). Current trends in telehealth applications to deliver social communication interventions for young children with or at risk for autism spectrum disorder. Current Developmental Disorders Reports 8, 15–23. 10.1007/s40474-020-00214-w 33072492 PMC7548134

[B38] Snodgrass, M. R., Chung, M. Y., Biller, M. F., Appel, K. E., Meadan, H., & Halle, J. W. (2017). Telepractice in speech-language therapy: The use of online technologies for parent training and coaching. Communication Disorders Quarterly, 38(4), 242–254. 10.1177/1525740116680424

[B39] SpeakEZ Theracare (n.d.). Home [Facebook Page]. Retrieved July 29, 2021 from https://www.facebook.com/SenseAntiguanSLP/

[B40] Stredler-Brown, A. S. (2017). Examination of early intervention delivered via telepractice with families of children who are deaf or hard of hearing. Perspectives of the ASHA Special Interest Groups, 2(9), 25–42. 10.1044/persp2.SIG9.25

[B41] Stoner, J. B., Meadan, H., & Angell, M. E. (2013). A model for coaching parents to implement teaching strategies with their young children with language delay or developmental disabilities. Perspectives in Language Learning and Education, 20(3), 112–119. 10.1044/lle20.3.112

[B42] Sussman, F. (1999). More than words: Helping parents promote communication and social skills in children with autism spectrum disorder. The Hanen Centre.

[B43] Van Balkom, H., Verhoeven, L., Van Weerdenburg, M., & Stoep, J. (2010). Effects of parent-based video home training in children with developmental language delay. Child Language Teaching and Therapy, 26(3), 221–237. 10.1177/0265659009349978

[B44] Vismara, L. A., Young, G. S., & Rogers, S. J. (2012). Telehealth for expanding the reach of early autism training to parents. Autism Research and Treatment, 2012, 121878. 10.1155/2012/121878 23227334 PMC3512210

[B45] Wright, C. A., & Kaiser, A. P. (2017). Teaching parents enhanced milieu teaching with words and signs using the Teach-Model-Coach-Review model. Topics in Early Childhood Special Education, 36(4), 192–204. 10.1177/0271121415621027

[B46] Weitzman, E. (2013). More than words – The Hanen program for parents of children with autism spectrum disorder: A teaching model for parent-implemented language intervention. Perspectives in Language Learning and Education, 20(3), 96–111. 10.1044/lle20.3.86

